# The Role of Cytology, Histology and Molecular Pathology in the Diagnostic Process of Thyroid Nodules

**DOI:** 10.3390/cancers18111814

**Published:** 2026-06-01

**Authors:** Mathilde Ribeiro, Sule Canberk, Massimo Bongiovanni

**Affiliations:** 1 Faculty of Medicine, University of Porto, 4200-319 Porto, Portugal; up202305227@edu.med.up.pt (M.R.); sulecanberk@med.up.pt (S.C.); 2 RISE-Health, Department of Pathology, Faculty of Medicine, University of Porto, 4200-319 Porto, Portugal; 3 Unilabs Pathology, 1003 Lausanne, Switzerland

**Keywords:** thyroid, fine-needle aspiration cytology, The Bethesda System for Reporting Thyroid Cytopathology, histological classification, diagnostic accuracy, molecular cytopathology

## Abstract

In recent years, thyroid nodule diagnosis and classification have been updated by two major developments: the fifth edition of the World Health Organization (WHO) Classification of Endocrine and Neuroendocrine Tumors classification 2022), which includes thyroid neoplasms, and the third edition of The Bethesda System for Reporting Thyroid Cytopathology (TBSRTC) (2023). Importantly, these classification systems converge in terminology, improving clinicopathologic correlation between fine-needle aspiration cytology (FNAC) and surgical pathology and supporting more consistent risk-based management. In parallel, molecular testing has assumed a growing role, particularly for indeterminate FNA categories, as reflected in contemporary practice recommendations. Moreover, the 2025 American Thyroid Association guidelines recalibrate postoperative risk stratification in differentiated thyroid cancer by reasserting histopathology as the cornerstone of clinical decision-making. In this review, we summarize key updates in thyroid FNAC reporting, histopathologic classification, and the practical application of molecular pathology for the evaluation and management of thyroid nodules.

## Simple Summary

The updated cytologic (The Bethesda System for Reporting Thyroid Cytopathology) and histologic (World Health Organization Classification of Endocrine and Neuroendocrine Tumors) classifications of thyroid neoplasms, together with the expanding use of molecular tests in thyroid nodules, have enhanced preoperative malignancy risk stratification and optimized patient management. The 2025 American Thyroid Association guidelines further refine postoperative risk stratification in differentiated thyroid carcinoma by integrating histologic classification and tumor histotype, underscoring the central role of histology in assessing recurrence risk, predicting radioactive iodine responsiveness, and tailoring follow-up strategies.

## 1. Introduction

The Bethesda System for Reporting Thyroid Cytopathology (TBSRTC) is a categorization system for providing uniform and standardized diagnosis in thyroid cytology that has proven its efficiency over the years [1]. It is widely used all over the world and has now reached its third edition, published in 2023. It has improved communication between cytopathologist and doctors implicated in the management of thyroid nodules to improve care of patients with thyroid diseases. The quantity of literature production and data over the years since its first edition in 2007 has permitted us to refine and ameliorate its purposes: to provide reliable diagnostic criteria associated with robust risk-of-malignancy rates for each diagnostic category and propose management actions based on scientific evidence [2]. Contemporarily, in 2023, the World Health Organization (WHO) published the fifth edition of the Classification of Tumors of the Endocrine and Neuroendocrine Systems (which also include the thyroid) used at the histological levels [3]. Although some contextual differences in application remain, both TBSRTC and the WHO use the same terminology in terms of definition of categories, increasing the diagnostic yield of cytology and understanding of cytological terminology. The indication on the use of molecular pathology was refined and reinforced over the diagnostic categories, helping to provide better management information based on amelioration of the risk of malignancy (ROM). The third edition of TBSRTC and the fifth edition of the WHO histological classification of thyroid tumors was adopted also by the American Thyroid Association (ATA) 2023 and 2025 guidelines for managing thyroid nodules [4].

This article is intended as a narrative review based on the authors’ personal experience, international guidelines for the management of patients with thyroid nodules, and the most recent literature on innovative technologies. It explains how the new cytological and histological classifications can improve diagnostic accuracy, how the application of molecular testing in thyroid lesions can enhance the diagnostic yield of surgery and assist clinicians in selecting the most appropriate therapeutic options, and what the future directions are for an integrated (morphology, molecular analysis, imaging and artificial intelligence) approach to the management of patients with thyroid nodules.

## 2. Management of Thyroid Nodules in Children

The third edition of TBSRTC also incorporates pediatric-specific considerations by providing pediatric ROM estimates for each diagnostic category [5]. While most pediatric nodules are benign, multiple cohorts have reported a higher malignancy rate in children than in adults, with substantial inter-study variability (Table 1) [6]. Pediatric differentiated thyroid carcinoma (DTC) also commonly presents with more advanced locoregional disease (including nodal involvement), yet outcomes are frequently favorable with appropriate risk-adapted therapy [5,6,7,8]. Importantly, in children, cytology-linked risk estimates are less extensively explored than in the adult literature. Although TBSRTC provides pediatric-focused considerations and acknowledges higher pretest probability in children, category-specific ROMs in pediatrics are derived from comparatively smaller series, are more sensitive to case-mix and referral patterns, and may not be directly interchangeable with adult reference ranges. In addition, the transferability of adult-derived refinements, such as proposed subtyping within indeterminate categories (e.g., AUS subgroups), to pediatric practice remains insufficiently validated; whether such subcategories reproducibly stratify ROM or meaningfully alter management in children has not been conclusively demonstrated across large, multi-institutional pediatric datasets. These limitations are reinforced by a recent multi-institutional PEDIMAP analysis, which included 363 pediatric and young-adult patients (0–25 years) managed surgically across four international centers with available somatic molecular profiling [7]. In that cohort, long-term outcome classified by ATA response-to-therapy criteria was not significantly stratified by TBSRTC cytology category (*p* = 0.164), supporting the view that adult-optimized cytomorphologic risk groupings may have reduced prognostic discrimination in the pediatric context. By contrast, oncogenic driver identity provided outcome-relevant biological stratification independent of age: RET fusions, NTRK1/3 fusions, and BRAF V600E were associated with significantly higher odds of a non-excellent response after adjustment, whereas RAS-mutant tumors showed a favorable trend and DICER1-mutant cases achieved excellent outcomes in the modeled cohort. Consequently, pediatric thyroid nodule management often requires more clinic–pathologic integration than is captured by cytology category alone (ultrasound risk, age, family history, radiation exposure, syndromic context, and institutional prevalence). Pediatric-specific management pathways for thyroid nodules are also less uniform than what is suggested in adult algorithms; in many settings, management of indeterminate (“grey-zone”) cytology in children is guided by a combination of pediatric thyroid cancer guidance, local multidisciplinary practice, and individualized risk tolerance rather than a single universally adopted pediatric nodule algorithm.

From a technical and interpretive standpoint, pediatric-specific pitfalls should be anticipated. Aspirates may inadvertently sample adjacent thymic tissue or thymic hyperplasia, which can mimic lymphoid proliferations if clinical and imaging findings are not carefully integrated. When feasible, rapid on-site evaluation (ROSE) is particularly valuable in children to optimize adequacy, reduce nondiagnostic rates, and minimize repeat procedures.

## 3. The Bethesda System for Reporting Thyroid Cytopathology

The detection of thyroid nodules has been steadily increasing due to the widespread use of ultrasonography (US) and the incorporation of whole-body computed tomography (CT) scans in the follow-up of patients with malignancies. By 2022, their prevalence had reached approximately 25%. However, the overall malignancy rate remains relatively low, ranging between 4% and 6.5%, as many thyroid nodules are benign [9]. The refinement of diagnostic criteria in thyroid ultrasonography, particularly through the application of Thyroid Imaging Reporting and Data Systems (TI-RADSs), has contributed to reducing the number of FNAC procedures, especially in nodules deemed benign on imaging [10,11]. Furthermore, the improvement of cytological diagnostic criteria, in combination with the growing use of molecular testing, has significantly decreased the proportion of indeterminate diagnoses—a long-standing challenge in thyroid FNAC. Recent data have demonstrated that ultrasound-guided FNAC achieves an overall diagnostic sensitivity of 72% and a specificity of 96%, with positive and negative predictive values of 93% and 75%, respectively, thus showing high diagnostic accuracy [12]. The third edition of TBSRTC maintains six diagnostic categories, as in the first edition: nondiagnostic, benign, atypia of undetermined significance (AUS), follicular neoplasm (FN), suspicious for malignancy (SM), and malignant (M) [1,13]. The corresponding ROM and recommended management for each category are summarized in Table 2. Notably, the terminology has been simplified to minimize ambiguity: categories previously termed AUS/FLUS and FN/SFN are now designated simply as AUS and FN, respectively. The ROM associated with each diagnostic category has been refined based on large-scale literature-derived data to better reflect contemporary findings. Institutions are encouraged to calculate their own ROM, as these values may vary according to regional patterns and population-specific factors [1].

### 3.1. Nondiagnostic (ND) Category

The first step in the cytologic assessment of a thyroid nodule is to determine specimen adequacy—whether the sample is representative of the lesion and contains enough well-preserved follicular cells to allow a reliable interpretation. The adequacy criterion of at least six groups of well-visualized follicular cells, each composed of a minimum of ten cells, on one or more slides has been retained in the current edition of TBSRTC. The overall ROM for ND specimens remains relatively high; therefore, repeat aspiration is recommended to achieve more precise characterization. Notably, the latest edition places no specific time interval before a repeat FNAC can be performed. Specimens consisting exclusively of cystic fluid with macrophages—so called “cyst content only” cases—continue to be classified as ND. The clinical interpretation of these findings should rely heavily on sonographic and clinicopathologic correlation. In the absence of suspicious sonographic features (e.g., mural nodularity or solid projections) and in a non-worrisome clinical context (no personal or family history of thyroid malignancy), the FNAC result can be managed as cytologically benign.

### 3.2. Benign Category

**Figure 1:**
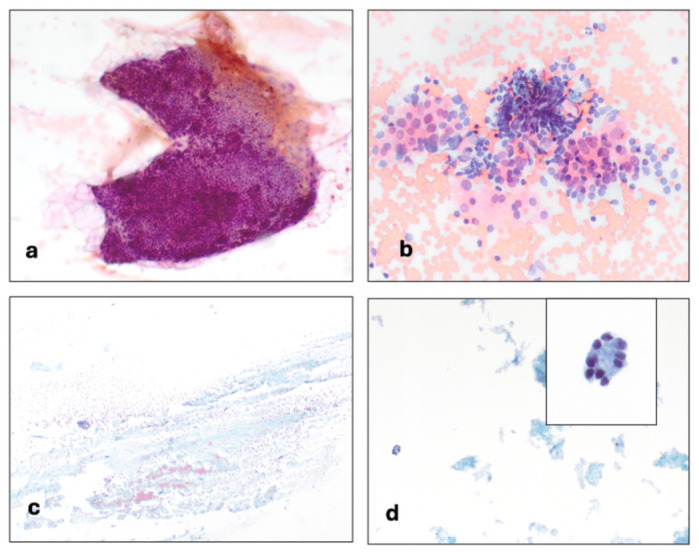
Examples of the benign and atypia of undetermined significance (AUS) cytological diagnoses. (**a**) Benign diagnosis: Large group of follicular cells arranged in macrofollicular structures filled with abundant colloid and no nuclear atypia (Papanicolaou stain, original magnification: 10×). (**b**) Benign diagnosis: Abundant oncocytes and lymphocytes in this aspiration of a patient with Hashimoto’s thyroiditis and a nodular lesion discovered during ultrasound examination. The diagnosis was: benign, consistent with Hashimoto’s thyroiditis (Papanicolaou stain, original magnification: 20×). (**c**) Benign diagnosis: Abundant colloid on the background of the smear with almost absence of thyrocytes. Although the criterion of 6 or more groups with at least 10 follicular cells per cluster has not been reached in this aspiration (needed for adequacy), this is a colloidal nodule and thus considered benign. This is an exception to the recommended cellular quantity adequacy criteria (Papanicolaou stain, original magnification: 4.5×). (**d**) AUS diagnosis, atypia-other: Scant cellularity in this specimen containing only a few follicles (<6 groups of follicles) arranged in a small microfollicular structure (inset) defining architectural atypia (Papanicolaou stain, original magnification: 10×).

FNAC demonstrates excellent diagnostic performance in identifying benign thyroid lesions, including follicular nodular disease, Hashimoto’s thyroiditis, and other benign proliferations. The associated ROM in this category is low, underscoring the high negative predictive value (NPV) of thyroid cytology for benign processes. Clinically, patients with benign results can be safely followed through routine observation and periodic ultrasound evaluation, unless new suspicious features arise. Cytologically, benign aspirates are characterized by abundant colloid and follicular epithelial cells arranged in large macrofollicles showing colloid distension. The follicular cells display uniform, round nuclei with smooth contours and lack significant atypia. In cases of thyroiditis, a mixed lymphoid component may be present and mild reactive nuclear atypia can be observed, consistent with inflammatory background changes (Figure 1).

### 3.3. Atypia of Undetermined Significance (AUS)

The marked interobserver variability in the use of this diagnostic category indicates that it has often functioned as a repository for thyroid FNAC cases that are not clearly benign or malignant, thereby diluting its clinical utility. To prevent overuse and to preserve the high predictive value of cytology for guiding management, it is generally recommended that AUS diagnoses should not exceed approximately 7–10% of all thyroid FNAC cases in a given practice. In addition to calculating the ROM, an important quality-control metric is the AUS-to-malignant ratio for each cytopathologist, which should ideally be ≤3 [14,15]. A major refinement introduced in the third edition of TBSRTC is the subdivision of AUS into two subgroups: AUS with nuclear atypia, associated with a higher ROM, and AUS-other, which encompasses architectural, oncocytic, inflammatory, and not-otherwise-specified atypia and is associated with a lower ROM. AUS with nuclear atypia includes cases in which the nuclear changes raise concern but are quantitatively or qualitatively insufficient for a definitive malignant or suspicious for malignancy diagnosis, often due to scant cellularity or suboptimal fixation and staining. A practical challenge is determining the upper threshold of acceptable nuclear atypia before upgrading to a higher-risk category. Some authors have proposed a semiquantitative nuclear scoring system that evaluates nuclear size and shape, membrane irregularities, and chromatin features, assigning a score of 0 or 1 to each parameter (total score 0–3), with nuclear atypia considered present only when the total score is 2 or 3. In routine practice, however, such scoring systems may be difficult to implement in AUS cases because the atypical nuclei are often focal and the specimens paucicellular and suboptimally preserved. Architectural atypia, by contrast, is defined by the presence of limited microfollicular or trabecular groups of cytologically bland thyrocytes, or follicular cells arranged in a streaming pattern reminiscent of a comet tail. Several studies have attempted to further subcategorize AUS to refine ROM estimates and better tailor clinical recommendations, consistently showing that nuclear atypia (in both non-oncocytic and oncocytic cells) carries a substantially higher ROM, reported in some series to be as high as 48% (Table 3). The introduction of the AUS category has improved the overall diagnostic performance of thyroid FNAC by reducing the number of false-negative benign diagnoses and increasing the proportion of cases correctly classified as “suspicious for malignancy” or “malignant.” A prudent approach in AUS cases is to perform at least one repeat FNAC before proceeding to more costly or technically demanding investigations, recognizing that molecular testing may be limited by low cellular yield in this setting. On repeat aspiration, most AUS cases are reclassified into the benign category, as increased cellularity and improved fixation and staining allow more confident interpretation. When AUS is confirmed on a second FNAC, the ROM may rise to approximately 30%, comparable to that of the follicular neoplasm category. Current ATA guidelines recommend that AUS results be interpreted in conjunction with ultrasound risk stratification and, when appropriate, adjunctive molecular testing to optimize patient management (Figure 1) [4,9,16,17,18,19,20,21].

### 3.4. Follicular Neoplasm (FN)

**Figure 2:**
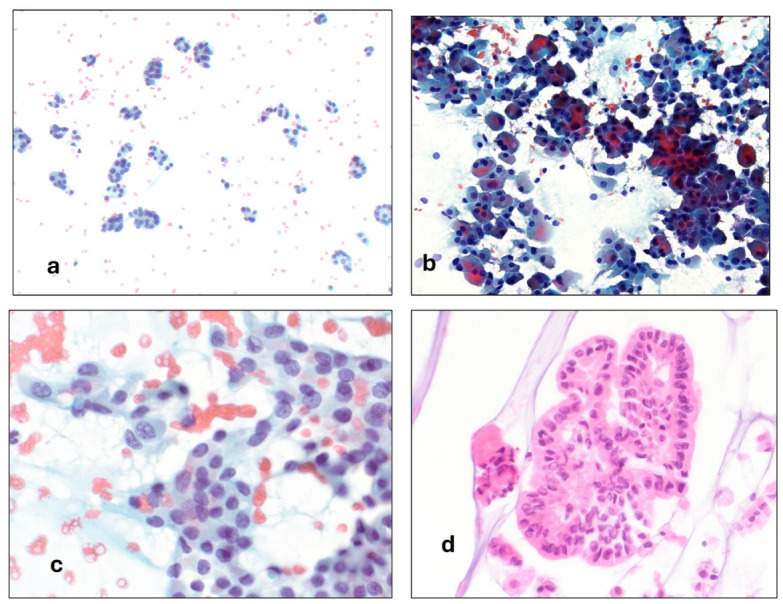
Examples of the follicular neoplasm, suspicious for malignancy and malignant diagnoses. (**a**) Follicular neoplasm diagnosis: A quite hypercellular smear composed almost exclusively of microfollicular structures. Malignancy risk can be refined by a molecular test; for final diagnosis, surgery and histopathological examination are required: follicular adenoma if no capsular and/or vascular invasion is detected vs. follicular carcinoma if capsular and/or vascular invasion are found (Papanicolaou stain, original magnification: 10×). (**b**) Follicular neoplasm, oncocytic type diagnosis: A hypercellular smear composed exclusively of oncocytes with large and abundant eosinophilic cytoplasm and black nuclei. In this case, as in the previous one, malignancy risk can be refined by a molecular test; for final diagnosis, surgery and histopathological examination is required (Papanicolaou stain, original magnification: 20×). (**c**) Suspicious for malignancy diagnosis: A pseudo-papillary group of thyrocytes with severe nuclear atypia, insufficient for a definitive diagnosis of papillary carcinoma (Papanicolaou stain, original magnification: 10×). (**d**) Malignant diagnosis: Well-defined papillary structures surrounded by thyrocytes with frank nuclear atypia and nuclear pseudo-inclusions (cell block, hematoxylin and eosin stain, original magnification: 40×).

The primary objective of this diagnostic category is to identify follicular-patterned lesions that carry a clinically significant risk, including follicular thyroid carcinoma (FTC), its oncocytic counterpart (oncocytic thyroid carcinoma, OTC), and follicular variant papillary thyroid carcinoma (FV-PTC). This category includes follicular neoplasm (FN) and its oncocytic subtype, oncocytic follicular neoplasm (OFN). The overall frequency of FN/OFN diagnoses among all TBSRTC categories is relatively low, generally reported in the range of approximately 1% to 12%. Morphologically, FN/OFN aspirates are at least moderately to markedly cellular and are composed predominantly of follicular epithelial cells arranged in microfollicular, trabecular, and/or crowded three-dimensional groups, often with variable degrees of papillary-type nuclear atypia or diffuse oncocytic change. When a preparation contains an admixture of clearly benign-appearing macrofollicular epithelium and the above-described follicular lesion, it may be more appropriate to render a benign diagnosis rather than FN, particularly in the presence of abundant colloid. OFN may exhibit a wide spectrum of nuclear changes. Limited papillary-type nuclear features may be acceptable within OFN; however, when such atypia is pronounced, the diagnosis should be upgraded to suspicious for malignancy or malignant, depending on the extent and quality of the changes. Nuclear enlargement and anisokaryosis are relatively common in oncocytic lesions and should not be overinterpreted as evidence of a poorly differentiated component, to avoid an unwarranted malignant diagnosis. When oncocytic proliferation is accompanied by a prominent lymphocytic infiltrate, Hashimoto’s thyroiditis should be strongly considered and a benign interpretation favored. The ROM for the FN category is typically in the range of 15–30%, reflecting the fact that FNAC serves as a screening tool for follicular-patterned neoplasms rather than a definitive discriminator between adenoma and carcinoma. Consequently, patients usually require further risk stratification with molecular testing and/or diagnostic surgery (typically lobectomy) to determine whether they harbor a low-risk neoplasm or an overt carcinoma. Molecular testing may either lower or increase the estimated ROM in individual cases, as discussed in dedicated molecular pathology sections in recent reviews. Importantly, if the final histologic examination discloses a benign follicular adenoma after an FN cytologic diagnosis, this does not necessarily imply a false-positive cytologic interpretation. Distinguishing benign from malignant follicular neoplasms requires demonstration of capsular and/or vascular invasion, which can only be assessed on a surgically resected specimen, most often a diagnostic lobectomy (Figure 2) [22].

### 3.5. Suspicious for Malignancy (SM)

Cases that lack the full spectrum of cytologic criteria required for a definitive malignant diagnosis, or in which malignant features are present in only a limited proportion of cells, are classified as SM. The ROM for this category is high, typically ranging from 67% to 83%, and thus cytology again functions as a diagnostic test in this setting. Most SM cases represent papillary thyroid carcinoma (PTC) in which one or more key diagnostic feature, such as well-formed papillary structures, intranuclear pseudo-inclusions, or psammoma bodies, are incomplete or only focally expressed. In addition, cases suspicious for medullary thyroid carcinoma (MTC) or lymphoma, in which ancillary techniques such as immunocytochemistry are not available or not feasible, may also be assigned to this category. Importantly, SM is not intended for lesions suspicious for follicular carcinoma, which are follicular-patterned and are more appropriately classified in the FN category rather than as SM or malignant. Recommended management usually involves further risk stratification with molecular testing and/or diagnostic lobectomy. When available, intraoperative frozen section examination may assist in determining the extent of surgery (lobectomy versus total thyroidectomy and possible lymph node dissection), although its utility is limited in some tumor types. The overarching aim of this category is to avoid overcalling lesions that could be managed more conservatively, thereby preserving the high positive predictive value (PPV) of cases formally diagnosed as malignant. The recent reclassification of selected thyroid tumors as low-risk neoplasms, such as noninvasive follicular thyroid neoplasm with papillary-like nuclear features (NIFTP), requires cytopathologists to apply stricter criteria before rendering a malignant diagnosis (Figure 2).

### 3.6. Malignant (M)

The M category is reserved for cases in which established cytologic criteria for a specific malignant entity, such as PTC, MTC, lymphoma, poorly differentiated carcinoma, or anaplastic (undifferentiated) carcinoma, are unequivocally fulfilled, resulting in a very high ROM (approximately 97–100%). For certain diagnoses, particularly MTC and lymphoma, immunocytochemical staining on direct smears or cell block material is strongly recommended to confirm the interpretation, given the major prognostic and therapeutic implications. In suspected MTC, correlation with clinical and biochemical findings, including elevated serum calcitonin and carcinoembryonic antigen (CEA), can support a positive diagnosis even when immunostains are not readily available. In cases suggestive of lymphoma, flow cytometric immunophenotyping is highly informative, allowing demonstration of a monoclonal lymphoid population and precise subclassification when feasible (Figure 2).

## 4. Use of Molecular Testing in Thyroid FNAC

Molecular testing has become an important adjunct in the evaluation of thyroid nodules with indeterminate FNAC results, and the third edition of TBSRTC explicitly discusses its role, particularly for AUS, FN, and (in selected contexts) SFM. In contemporary practice recommendations from ATA and other professional societies, molecular testing is positioned as a risk-refinement tool rather than a replacement for cytomorphology, ultrasound risk stratification, and clinicopathologic correlation. Several analytical strategies can be applied to FNAC-derived material. These range from targeted single-gene assays or small focused panels (still common in some European workflows) to larger next-generation sequencing panels and classifier-based approaches incorporating DNA, RNA, and/or microRNA profiles (more frequently used in the United States). While platforms differ, the clinical intent is similar: to refine the post-test probability of malignancy in nodules that are cytologically indeterminate, thereby informing decisions between surveillance, diagnostic lobectomy, and more definitive surgery. The clinical utility of any molecular result depends on the pretest probability (local prevalence and ultrasound risk), the cytology category, and the technical adequacy of the specimen; therefore, institutions should interpret molecular results within an explicitly Bayesian framework and, where possible, validate local performance. The third edition of TBSRTC describes molecular results in terms of three clinically meaningful strata: low, intermediate, and high molecular probability of cancer [1]. In practice, low-probability results function as “rule-out” evidence that may support observation in appropriately selected patients when concordant with low-risk ultrasound and clinical features. High-probability results function as “rule-in” evidence that strengthens the rationale for surgical management. Intermediate-probability results remain the most challenging category; as with indeterminate cytology itself, these results often do not eliminate the need for surgery, and management should be individualized based on ultrasound pattern, patient factors, institutional ROM, and feasibility of surveillance. Importantly, molecular testing should be ordered only when the result is expected to change management (e.g., surveillance versus diagnostic surgery, or extent of initial surgery).

Briefly, among the larger panels used in the United States, both ThyroSeq and Veracyte’s Afirma Genomic Sequencing Classifier (GSC) are next-generation-sequencing-based platforms [23,24,25]. Afirma GSC is an RNA-based genomic classifier that integrates gene expression data and sequencing-derived features to categorize indeterminate nodules as either “benign” or “suspicious.” Its design primarily emphasizes rule-out performance. In surgically confirmed nodules, Afirma GSC demonstrates high sensitivity (~94%) and a high negative predictive value (NPV) (~96%). When test-negative nodules that do not undergo surgery are assumed to be benign—reflecting real-world clinical practice—its specificity increases substantially (to ~86%), and the NPV approaches 99%. These characteristics make Afirma GSC particularly valuable for avoiding unnecessary hemithyroidectomy [26,27]. ThyroSeq v3 analyzes more than 100 genetic alterations, including point mutations, gene fusions, copy number alterations, and other genomic markers. As a rule-out test, it demonstrates a sensitivity of approximately 96% and an NPV of around 93% in surgically confirmed nodules. When unoperated test-negative nodules are included as true negatives, specificity increases to approximately 83% and the NPV rises to 99%. ThyroSeq can also identify high-risk mutations, such as TERT promoter mutations, TP53, and BRAF V600E, which may carry prognostic and, in some cases, therapeutic implications [28,29]. Despite this, its specificity in surgically confirmed nodules remains relatively low (~40%), similar to Afirma GSC, meaning that a “suspicious” result does not reliably confirm malignancy. While Afirma GSC and ThyroSeq v3 are best suited for avoiding unnecessary surgery (rule-out tests), focused panels are generally smaller, mutation-centered assays that trade breadth for cost efficiency and accessibility. These assays are primarily useful as rule-in tests, helping to confirm malignancy when a known driver mutation is detected. Such panels typically include BRAF V600E (associated with classic papillary thyroid carcinoma), RAS mutations, TERT promoter mutations (associated with more aggressive disease behavior), and common fusions such as RET/PTC and PAX8/PPARG. They demonstrate high specificity when a driver mutation is identified; however, their sensitivity and NPV are substantially lower because many malignant nodules do not harbor these specific alterations [30,31].

A specific caveat applies to oncocytic-patterned lesions. Oncocytic morphology on FNA can reflect biologically heterogeneous entities, and oncocytic tumors often show molecular architectures that differ from classic RAS-like follicular-patterned lesions, including prominent copy number alterations and mitochondrial-DNA-related changes in subsets. In a recent comparative multi-omics study of oncocytic cell tumors, true oncocytic cell tumors formed a distinct molecular cluster compared with mitochondrion-rich neoplasms, showing that “oncocytic morphology” is not a single biologic category [32]. The dominant altered programs in oncocytic cell tumors involved epigenetic regulation, oxidative/heme-related pathways, and protein/mitochondrial biogenesis. Practically, these data support a cautious approach to interpreting molecular results in oncocytic-predominant AUS/FN nodules: results may still be clinically useful for risk refinement, but they should be interpreted carefully. Beyond indeterminate nodule triage, molecular profiling has an additional role in established thyroid malignancies—particularly aggressive, widely invasive, metastatic, or unresectable disease—where identification of actionable alterations can inform systemic therapy selection. Depending on tumor type and clinical scenario, relevant biomarkers may include kinase alterations (e.g., BRAF, RET, NTRK, and ALK), as well as broader genomic features such as tumor mutational burden and mismatch repair/microsatellite instability status when clinically indicated. In these settings, molecular testing is primarily therapeutic and prognostic rather than diagnostic, and results should be discussed in a multidisciplinary framework.

## 5. Core-Needle Biopsy

**Figure 3:**
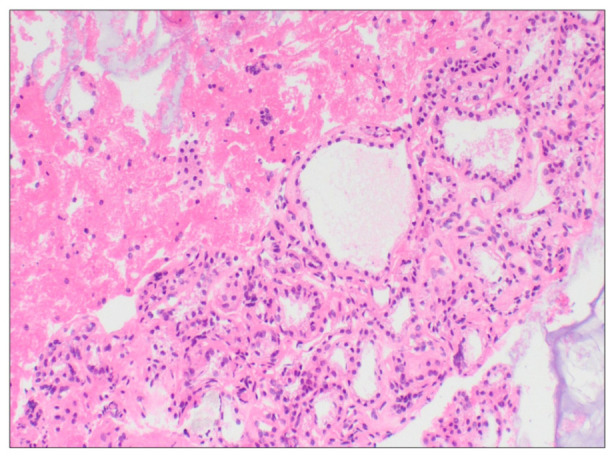
A core-needle biopsy performed following a diagnosis of atypia of undetermined significance (AUS) on fine-needle aspiration: it is composed of a mixed population of macrofollicular and microfollicular structures, with no nuclear atypia, and has been diagnosed as benign. No capsular component was visible on the biopsy (hematoxylin and eosin stain, original magnification: 20×).

Core-needle biopsy (CNB) should be positioned as a problem-solving, tissue-acquisition procedure in thyroid diagnostics, reserved for scenarios in which additional histologic material is likely to change immediate management. Its highest-impact indication is a rapidly enlarging thyroid mass with clinical and imaging suspicion for anaplastic thyroid carcinoma (ATC), because CNB can simultaneously (i) secure diagnostically robust material for morphology and immunohistochemistry to separate ATC from key mimics (particularly lymphoma, metastatic carcinoma, and medullary thyroid carcinoma), and (ii) provide sufficient tissue for urgent molecular testing with direct therapeutic implications. In this setting, rapid assessment of BRAF V600E has become especially relevant because BRAF/MEK-targeted therapy is an established option for BRAF V600E-mutant ATC, and contemporary guidance emphasizes expeditious molecular profiling at diagnosis to identify actionable alterations. CNB is also valuable when lymphoma or metastasis is suspected and a definitive diagnosis requires immunophenotyping or lineage-defining immunohistochemistry, or when repeated FNA is ND yet sonographic/clinical risk remains high and additional tissue is required for ancillary studies. By contrast, CNB has limited ability to resolve follicular-patterned neoplasms because malignancy in follicular carcinoma depends on demonstrating capsular and/or vascular invasion across the tumor–capsule interface, which cannot be reliably assessed on limited core samples; therefore, even when CNB suggests a follicular neoplasm, diagnostic lobectomy often remains necessary for definitive classification. Finally, if CNB is used to support molecular testing, results should be interpreted in a pretest-probability framework (cytology category, ultrasound pattern, and local prevalence), recognizing that many alterations are not fully specific for adenoma versus carcinoma, while certain alterations (e.g., BRAF V600E in an appropriate morphologic context) carry strong diagnostic and, in ATC, therapeutic relevance (Figure 3) [33,34].

## 6. New Concepts in the WHO Thyroid Tumor Classification

The fifth edition of the WHO Classification of Endocrine and Neuroendocrine Tumors reflects progress in understanding thyroid tumor biology and, where evidence is mature, incorporates molecular correlates that are relevant to diagnosis and clinical behavior [3,35]. For thyroid cytology practice, the most important consequences are improved terminology clarity and a behavior-based framing of selected tumor groups that support risk-adapted management and reduce the likelihood of overtreatment driven by diagnostic labels. The WHO promotes harmonized terminology that improves cytology–histology communication. As an example, in thyroid pathology reporting, oncocytic is preferred over Hürthle, and this convergence is also relevant to FNAC reporting because it reduces ambiguity at the interface between cytology categories and the corresponding histopathologic entities. A key behavior-based refinement is the clearer separation of an indolent borderline spectrum among encapsulated follicular patterned lesions. This space includes non-invasive follicular thyroid neoplasm with papillary-like nuclear features, follicular tumor of uncertain malignant potential and well-differentiated tumor of uncertain malignant potential, and hyalinizing trabecular tumor. The essential concept is not the absence of molecular alterations but rather that when strict histologic criteria are met, these lesions show very low rates of clinically significant spread. This has direct implications for cytology, because FNAC cannot assess capsular or vascular invasion or confirm noninvasiveness, and therefore cytology should avoid definitive carcinoma wording in settings that may ultimately fall into these low-risk categories. This behavior-oriented approach is one of the factors supporting stricter thresholds in the third edition of TBSRTC. Hyalinizing trabecular tumors warrant specific mention because they may mimic PTC on cytology and can occasionally enter the differential diagnosis of other malignant entities [36]. When material is sufficient for ancillary testing, identification of a GLIS family rearrangement provides strong support for this diagnosis and can be particularly helpful when morphology is equivocal. Tumors of uncertain malignant potential are uncommon and, in routine cytology practice, usually fall within an FN type interpretation because invasion cannot be assessed [37]. At the aggressive end of follicular-cell-derived carcinomas, the WHO emphasizes a high-grade non-anaplastic spectrum that includes differentiated high-grade thyroid carcinoma and poorly differentiated thyroid carcinoma. These entities have outcomes intermediate between well-differentiated thyroid carcinoma and anaplastic thyroid carcinoma. Cytology has inherent limits in grading because key histologic parameters such as mitotic activity and tumor necrosis may be underrepresented or difficult to quantify in many cytology preparations. Accordingly, cytology reports should, when possible, communicate the presence of high-grade features and prompt appropriate triage for ancillary testing and definitive management rather than attempting histologic grade assignment. The WHO also incorporates grade stratification principles for MTC using proliferative (Ki-67/Mib-1 immunostaining) and necrosis-based parameters that are typically assessed histologically. Although these metrics are not routinely measurable on cytology, their existence reinforces the importance of obtaining adequate material for immunohistochemical confirmatory testing, when MTC is morphologically suspected, because the diagnosis carries specific prognostic and therapeutic consequences. Further alignment between cytology and surgical pathology terminology is in progress. A WHO Head and Neck Cytology Reporting System is currently in development and is intended to provide a unified reporting framework across thyroid, salivary gland, and other head and neck sites. If implemented as planned, this framework may improve interoperability between cytology terminology and WHO-defined histopathologic entities while allowing organ-specific refinements, including expanded pediatric considerations, as evidence evolves.

## 7. Integration of Histopathology, Molecular Alterations, and the Tumor Microenvironment in Thyroid Cancer Progression

Although histopathology remains the cornerstone of thyroid cancer diagnosis and prognostication, recent advances—particularly those emerging from The Cancer Genome Atlas thyroid carcinoma study—have clearly demonstrated the close integration between histopathological features and molecular alterations. Classical histological patterns correspond to distinct molecular subtypes of papillary thyroid carcinoma (PTC) [38]. Tumors harboring BRAF mutations typically exhibit classical papillary architecture, nuclear clearing, and stromal fibrosis, reflecting hyperactivation of the MAPK signaling pathway. In contrast, RAS-mutated tumors more frequently display follicular growth patterns, encapsulation, and a more indolent cytological appearance. This genotype–phenotype correlation established the basis for the “BRAF-like” and “RAS-like” molecular dichotomy that now underpins much of the contemporary classification of thyroid tumors.

The biological progression from well-differentiated thyroid carcinoma to poorly differentiated thyroid carcinoma (PDTC) and anaplastic thyroid carcinoma (ATC) has been elucidated through seminal studies by James A. Fagin and Samuel A. Wells Jr [39], as well as by Irina Landa and colleagues [40]. These studies demonstrated that tumor dedifferentiation is driven by the sequential accumulation of high-risk molecular alterations, including TERT promoter mutations, TP53 mutations, PIK3CA alterations, and widespread copy number changes. Landa and colleagues further showed that PDTC and ATC exhibit transcriptomic signatures characterized by cell-cycle dysregulation, chromatin-remodeling defects, and activation of inflammatory and epithelial–mesenchymal transition (EMT) pathways. These molecular events closely correspond to the morphological features of dedifferentiation—including solid or trabecular growth patterns, tumor necrosis, and high mitotic activity—highlighting the biological continuity between histological morphology and molecular evolution.

Large-scale sequencing studies of advanced thyroid cancers, including the analysis of 779 tumors by Alexander Pozdeyev and colleagues, have reinforced these findings by demonstrating that TERT promoter mutations, TP53 alterations, and PI3K pathway activation are strongly associated with metastatic behavior, treatment resistance, and loss of differentiation [41]. Similarly, Tihana Ibrahimpasic and colleagues emphasized that PDTC represents a biologically intermediate entity, often harboring both RAS-like and BRAF-like alterations, and that its diagnosis benefits from the integration of histopathological criteria with molecular data [42]. These observations support the emphasis of the present manuscript on clinicopathological integration, particularly in indeterminate cytology categories in which morphology alone may not fully capture biological risk.

Immunohistochemical markers provide an additional layer of biological information that complements both cytological and molecular analyses and can be readily incorporated into routine practice. The Ki-67 proliferation index increases progressively from differentiated thyroid carcinoma to PDTC and ATC, reflecting enhanced proliferative activity. Aberrant p53 expression, corresponding to TP53 mutations, is a hallmark of dedifferentiated tumors and reflects underlying genomic instability. Similarly, PD-L1 expression, typically low in differentiated thyroid carcinomas, becomes markedly increased in aggressive variants, reflecting mechanisms of immune evasion and suggesting potential therapeutic relevance. In contrast, markers associated with EMT remain largely confined to research settings and are not routinely used in clinical practice [43,44].

The tumor microenvironment (TME), although still primarily investigated in the research setting, has emerged as a critical determinant of thyroid cancer aggressiveness. Classical PTC frequently demonstrates lymphocytic infiltration, sometimes associated with Hashimoto thyroiditis, which may confer a more favorable prognosis. By contrast, PDTC and ATC are characterized by a profoundly immunosuppressive microenvironment, with reduced infiltration by cytotoxic T lymphocytes and increased numbers of regulatory T cells and M2-polarized tumor-associated macrophages (TAMs). TAMs promote tumor invasion, angiogenesis, and immune suppression through the secretion of IL-10, TGF-β, and VEGF, and their density correlates with the presence of BRAF V600E and TERT promoter mutations. Furthermore, immune checkpoint molecules—including PD-L1, TIM-3, LAG-3, and CTLA-4—are upregulated in aggressive thyroid tumors, reflecting an exhausted immune microenvironment and identifying potential candidates for immunotherapy. Inflammatory signaling pathways, such as NF-κB and JAK/STAT, further promote EMT, dedifferentiation, and resistance to radioactive iodine therapy, thereby establishing a mechanistic link between chronic inflammation and morphological progression [45].

These molecular and microenvironmental insights also inform the interpretation of the diagnostic molecular platforms discussed earlier in this manuscript. The integration of broad next-generation sequencing assays, focused mutation panels with histopathology and TME characteristics provides a more comprehensive and clinically actionable understanding of thyroid cancer biology, consistent with the diagnostic and management principles outlined in the updated TBSRTC and WHO classifications.

## 8. Innovative Technologies in Thyroid Cytology and Histopathology

Recent technological advances are reshaping diagnostic practice in pathology. Digital pathology, artificial intelligence (AI), and imaging-based radiomics have emerged as powerful tools that enhance diagnostic accuracy, reproducibility, and workflow efficiency, while also opening new avenues for personalized risk stratification.

Digital pathology has expanded rapidly also in clinical settings. High resolution digitization of cytology and histology slides facilitates remote consultation and improves diagnostic accuracy in difficult cases. Digital cytology for thyroid FNAC achieves diagnostic concordance comparable to conventional microscopy, with additional advantages in image sharing and quantitative morphometric analysis [46]. Digital platforms also support computational pipelines for automated nuclear feature extraction, which is particularly relevant for categories such as AUS, where subtle nuclear changes influence risk stratification.

AI and deep learning have shown promising performance in thyroid FNAC interpretation. Convolutional neural networks (CNNs) trained on large datasets of digitized thyroid smears have achieved high accuracy in distinguishing benign from malignant nodules, in predicting Bethesda categories and identifying papillary thyroid carcinoma with sensitivities and specificities exceeding 90% [47,48]. AI-assisted quantification of nuclear irregularities, chromatin texture, and architectural patterns may help reduce interobserver variability, particularly in indeterminate categories such as AUS and FN. In future, multimodal AI systems that integrate cytology images with ultrasound features and molecular data could refine better malignancy risk and offer a multimodal approach to malignancy prediction [49].

Also, ultrasound-based radiomic signatures have been shown to predict malignancy, differentiate PTC variants, and correlate with molecular alterations such as BRAF V600E. CT and MRI radiomics have demonstrated utility in identifying aggressive phenotypes, including extrathyroidal extension and lymph node metastasis, particularly in PDTC and ATC. Several studies have proposed radiomic-based nomograms that integrate imaging features with clinical and cytological data, improving preoperative risk stratification beyond conventional TI RADS scoring [50,51].

## 9. Molecular Alterations and Targeted Therapeutic Implications in Thyroid Cancer

Specific molecular alterations (like BRAF V600E, RET fusions, NTRK fusions, and RAS mutations) can be translated into actionable therapeutic opportunities. For example, BRAF-mutated anaplastic and refractory differentiated thyroid carcinomas have shown meaningful responses to combined BRAF/MEK inhibition with dabrafenib and trametinib, as demonstrated in recent clinical studies [52]. Similarly, the development of highly selective RET inhibitors, such as selpercatinib and pralsetinib, has transformed the management of RET-mutated medullary thyroid carcinoma and RET-fusion-positive papillary thyroid carcinoma, with compelling results from the LIBRETTO 001 and ARROW trials [53]. The efficacy of NTRK inhibitors, including larotrectinib and entrectinib, in NTRK-fusion-positive thyroid cancers is also remarkable, supported by pooled analyses showing durable responses across tumor types [54]. Similarly, multikinase inhibitors such as lenvatinib and sorafenib in radioiodine refractory differentiated thyroid carcinoma, based on the SELECT and DECISION trials, have also shown efficacy.

Considering the role of immunotherapy in aggressive thyroid cancers, immune checkpoint inhibitors, including pembrolizumab and nivolumab, have shown activity in selected patients with ATC and PDTC, particularly in tumors with high PD L1 expression or elevated tumor mutational burden. Combination strategies—such as pairing BRAF/MEK inhibitors with immunotherapy—may offer synergistic benefit in highly aggressive disease [55].

## 10. Conclusions

**Figure 4:**
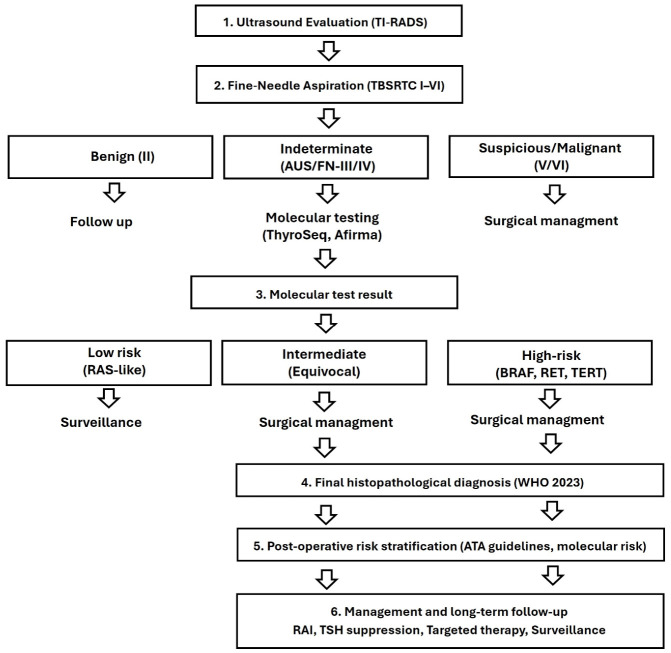
Integrated algorithm combining ultrasound evaluation, Bethesda cytology, molecular testing, histopathology and management.

The central message of this review is that thyroid nodule care is moving from isolated test-based decisions to integrated, risk-calibrated decision making (Figure 4). Ultrasound risk stratification reduces unnecessary sampling, thyroid FNAC remains the pivotal triage test, and the third edition of TBSRTC strengthens reproducibility by refining diagnostic thresholds and clarifying how indeterminate categories should be used and audited locally. Molecular testing has a defined and practical role when ordered selectively for indeterminate cytology and interpreted in a pretest probability framework, and its greatest value is achieved when results are explicitly mapped to management decisions rather than reported as standalone labels. Special contexts highlight the same principle: pediatric nodules require cautious extrapolation from adult ROM tables and benefit from deeper clinicopathologic integration; oncocytic-morphology-dominant patterned nodules require interpretive caution because morphology can represent biologically distinct classes, and core-needle biopsy is most clinically decisive when urgent tissue is needed to confirm aggressive disease or exclude key mimics and to enable actionable testing, particularly in suspected anaplastic carcinoma. At the histopathology interface, the WHO Classification of Endocrine and Neuroendocrine Tumors reinforces behavior-based concepts and clearer terminology that directly affect cytology thresholds, especially by discouraging overdiagnosis of borderline spectrum lesions and by clarifying high-grade tumor groupings that demand prompt triage. Looking forward, further convergence of language and risk logic is expected through the forthcoming WHO Head and Neck Cytology Reporting System, intended to harmonize cytology reporting across the thyroid and other head and neck organs while allowing organ-specific and pediatric refinements as evidence matures. Overall, the practical endpoint is consistent multidisciplinary communication, local ROM auditing, and patient-centered management choices that integrate imaging, cytomorphology, histology concepts, and molecular information when it changes care.

## Figures and Tables

**Table 1:** Comparison of the ROM in the pediatric and adult population (ref. [1]).

Diagnostic Category	ROM in Pediatric Population. Mean % (Range)	ROM in Adult Population. Mean % (Range)
Nondiagnostic	14 (0–33)	13 (5–20)
Benign	6 (0–27)	4 (2–7)
Atypia of Undetermined Significance	28 (11–54)	22 (13–30)
Follicular Neoplasm	50 (28–100)	30 (23–34)
Suspicious for Malignancy	81 (40–100)	74 (67–83)
Malignant	98 (86–100)	97 (97–100)

**Table 2:** The Bethesda System for Reporting Thyroid Cytopathology, 3rd edition, with associated risk of malignancy and proposed management (ref. [1]).

Diagnostic Category	Risk of Malignancy, Mean	Proposed Management
Nondiagnostic	13%	Repeat FNA with ultrasound guidance
Benign	4%	Clinical/sonographic follow-up
Atypia of Undetermined Significance	22%	Repeat FNA, molecular testing, diagnostic lobectomy, or surveillance
Follicular Neoplasm	30%	Molecular testing, diagnostic lobectomy
Suspicious for Malignancy	74%	Molecular testing, lobectomy or near-total thyroidectomy
Malignant	97%	Molecular testing, lobectomy or near-total thyroidectomy

Abbreviations. FNA: fine-needle aspiration.

**Table 3:** Differentiated risk of malignancy in the atypia of undetermined significance diagnostic category according to its subtype (ref. [20]).

AUS Subtype	Risk of Malignancy Estimated, Range	Proposed Management
AUS—Nuclear Atypia	36–48%	Repeat FNA, molecular testing, surgery
AUS—Other (Architectural)	10–20%	Repeat FNA, follow-up
AUS—Other (Oncocytic)	5–6%	Repeat FNA, follow-up (if there are no atypical nuclear features)
AUS—Other (NOS)	10–20%	Repeat FNA, second option

Abbreviations. AUS: atypia of undetermined significance; FNA: fine-needle aspiration.

## Data Availability

No new data were created or analyzed in this study.

## References

[1] Ali S.Z., Baloch Z.W., Cochand-Priollet B., Schmitt F.C., Vielh P., VanderLaan P.A. (2023). The Bethesda System for Reporting Thyroid Cytology.

[2] Ali S.Z., Baloch Z.W., Cochand-Priollet B., Schmitt F.C., Vielh P., VanderLaan P.A. (2023). The 2023 Bethesda System for Reporting Thyroid Cytopathology. Thyroid.

[3] Asa S.L., Baloch Z.W., Erickson L.A., International Agency for Research on Cancer (2025). WHO Classification of Tumours of Endocrine and Neuroendocrine Tumours.

[4] Ringel M.D., Sosa J.A., Baloch Z., Bischoff L., Bloom G., Brent G.A., Brock P.L., Chou R., Flavell R.R., Goldner W. (2025). 2025 American Thyroid Association Management Guidelines for Adult Patients with Differentiated Thyroid Cancer. Thyroid.

[5] Francis G.L., Waguespack S.G., Bauer A.J., Angelos P., Benvenga S., Cerutti J.M., Dinauer C.A., Hamilton J., Hay I.D., Luster M. (2015). Management Guidelines for Children with Thyroid Nodules and Differentiated Thyroid Cancer. Thyroid.

[6] Canberk S., Barroca H., Girão I., Aydın O., Uguz A., Erdogan K., Tastekin E., Bongiovanni M., Soares P., Máximo V. (2022). Performance of the Bethesda System for Reporting Thyroid Cytology in Multi-Institutional Large Cohort of Pediatric Thyroid Nodules: A Detailed Analysis. Diagnostics.

[7] Canberk S., Isaza A., Bojarsky M., Simplício M., Barroca H., Akkoyunlu S.Z., Günver G., Almeida I., Dello Iacovo F., Carillo A.M. (2026). The influence of age-independent somatic driver alterations on clinical outcomes in paediatric and young adult thyroid cancer. Eur. Thyroid J..

[8] Nies M., Vassilopoulou-Sellin R., Bassett R.L., Yedururi S., Zafereo M.E., Cabanillas M.E., Sherman S.I., Links T.P., Waguespack S.G. (2021). Distant Metastases from Childhood Differentiated Thyroid Carcinoma: Clinical Course and Mutational Landscape. J. Clin. Endocrinol. Metab..

[9] Bagis M., Can N., Sut N., Tastekin E., Erdogan E.G., Bulbul B.Y., Sezer Y.A., Kula O., Demirtas E.M., Usta I. (2024). A Comprehensive Approach to the Thyroid Bethesda Category III (AUS) in the Transition Zone Between 2nd Edition and 3rd Edition of The Bethesda System for Reporting Thyroid Cytopathology: Subcategorization, Nuclear Scoring, and More. Endocr. Pathol..

[10] Borges A.P., Antunes C., Caseiro-Alves F., Donato P. (2023). Analysis of 665 thyroid nodules using both EU-TIRADS and ACR TI-RADS classification systems. Thyroid Res..

[11] Campennì A., Barbaro D., Guzzo M., Capoccetti F., Giovanella L. (2020). Personalized management of differentiated thyroid cancer in real life—Practical guidance from a multidisciplinary panel of experts. Endocrine.

[12] Zulfa P.O., Iqhrammullah M., Zufry H. (2025). Diagnostic accuracy of preoperative ultrasonography-guided fine-needle aspiration biopsy in distinguishing malignancy in large thyroid nodules: A systematic review, meta-analysis, and meta-regression. Narra J..

[13] Luis R., Thirunavukkarasu B., Jain D., Canberk S. (2024). Welcoming the new, revisiting the old: A brief glance at cytopathology reporting systems for lung, pancreas, and thyroid. J. Pathol. Transl. Med..

[14] Gokozan H.N., Dilcher T.L., Alperstein S.A., Qiu Y., Mostyka M., Scognamiglio T., Solomon J.P., Song W., Rennert H., Beg S. (2022). Combining molecular testing and the Bethesda category III:VI ratio for thyroid fine-needle aspirates: A quality-assurance metric for evaluating diagnostic performance in a cytopathology laboratory. Cancer Cytopathol..

[15] Krane J.F., Vanderlaan P.A., Faquin W.C., Renshaw A.A. (2012). The atypia of undetermined significance/follicular lesion of undetermined significance:malignant ratio: A proposed performance measure for reporting in The Bethesda System for thyroid cytopathology. Cancer Cytopathol..

[16] Guerreiro S.C., Tastekin E., Mourao M., Loureiro I., Eusebio R., Marques H.P., Oznur M., Caliskan C.K., Schmitt F.C., Bongiovanni M. (2023). Impact of the 3rd Edition of the Bethesda System for Reporting Thyroid Cytopathology on Grey Zone Categories. Acta Cytol..

[17] Slowinska-Klencka D., Popowicz B., Duda-Szymanska J., Klencki M. (2025). Thyroid Nodules with Nuclear Atypia of Undetermined Significance (AUS-Nuclear) Hold a Two-Times-Higher Risk of Malignancy than AUS-Other Nodules Regardless of EU-TIRADS Class of the Nodule or Borderline Tumor Interpretation. Cancers.

[18] Słowińska-Klencka D., Wysocka-Konieczna K., Woźniak-Oseła E., Sporny S., Popowicz B., Sopiński J., Kaczka K., Kuzdak K., Pomorski L., Klencki M. (2019). Thyroid nodules with Hürthle cells: The malignancy risk in relation to the FNA outcome category. J. Endocrinol. Investig..

[19] Smulever A., Pitoia F. (2021). Active surveillance in small cytological indeterminate thyroid nodules: A call to common sense?. Endocrine.

[20] Canberk S., Simplício M., Bongiovanni M., Rodrigues E., Capela J. (2025). Ten Key Questions on the 2023 Bethesda “AUS” Category: Practical Guidance for Surgeons, Endocrinologists, and Pathologists. Port. J. Surg..

[21] Ovčariček P.P., Campennì A., Bongiovanni M., Giovanella L. (2025). The management of cytologically indeterminate thyroid nodules in clinical practice: A contemporary perspective with focus on molecular imaging. Endocrine.

[22] Lametti A., Brimo F., Kanber Y., Caglar D., Auger M. (2025). Cytopathology of follicular and oncocytic follicular thyroid neoplasms: A Bethesda System perspective. Cancer Cytopathol..

[23] Dowell N., Begum S., Muzaffar J., Boelaert K., Nieto H. (2025). Comparing the diagnostic accuracy of Afirma GSC to ThyroSeq V3 in cytologically indeterminate thyroid nodules. Eur. Thyroid J..

[24] Vardarli I., Tan S., Görges R., Krämer B.K., Herrmann K., Brochhausen C. (2024). Diagnostic accuracy of Afirma gene expression classifier, Afirma gene sequencing classifier, ThyroSeq v2 and ThyroSeq v3 for indeterminate (Bethesda III and IV) thyroid nodules: A meta-analysis. Endocr. Connect..

[25] Silaghi C.A., Lozovanu V., Georgescu C.E., Georgescu R.D., Susman S., Năsui B.A., Dobrean A., Silaghi H. (2021). Thyroseq v3, Afirma GSC, and microRNA Panels Versus Previous Molecular Tests in the Preoperative Diagnosis of Indeterminate Thyroid Nodules: A Systematic Review and Meta-Analysis. Front. Endocrinol..

[26] Angell T.E., Heller H.T., Cibas E.S., Barletta J.A., Kim M.I., Krane J.F., Marqusee E. (2019). Independent Comparison of the Afirma Genomic Sequencing Classifier and Gene Expression Classifier for Cytologically Indeterminate Thyroid Nodules. Thyroid.

[27] Patel K.N., Angell T.E., Babiarz J., Barth N.M., Blevins T., Duh Q.Y., Ghossein R.A., Harrell R.M., Huang J., Kennedy G.C. (2018). Performance of a Genomic Sequencing Classifier for the Preoperative Diagnosis of Cytologically Indeterminate Thyroid Nodules. JAMA Surg..

[28] Chiosea S., Hodak S.P., Yip L., Abraham D., Baldwin C., Baloch Z., Gulec S.A., Hannoush Z.C., Haugen B.R., Joseph L. (2023). Molecular Profiling of 50 734 Bethesda III–VI Thyroid Nodules by ThyroSeq v3: Implications for Personalized Management. J. Clin. Endocrinol. Metab..

[29] Nikiforova M.N., Mercurio S., Wald A.I., Barbi de Moura M., Callenberg K., Santana-Santos L., Gooding W.E., Yip L., Ferris R.L., Nikiforov Y.E. (2018). Analytical performance of the ThyroSeq v3 genomic classifier for cancer diagnosis in thyroid nodules. Cancer.

[30] Nikiforov Y.E., Ohori N.P., Hodak S.P., Carty S.E., LeBeau S.O., Ferris R.L., Yip L., Seethala R.R., Tublin M.E., Stang M.T. (2011). Impact of mutational testing on the diagnosis and management of patients with cytologically indeterminate thyroid nodules: A prospective analysis of 1056 FNA samples. J. Clin. Endocrinol. Metab..

[31] Nikiforova M.N., Nikiforov Y.E. (2008). Molecular genetics of thyroid cancer: Implications for diagnosis, treatment and prognosis. Expert Rev. Mol. Diagn..

[32] Canberk S., Ferreira M., Da Cruz Paula A., Pereira L., Oliveira C., Osório H., Soares P., Máximo V. (2026). Unravelling the Tumourigenesis Mechanisms of Oncocytic Cell Tumours: Discoveries from a Comparative Omics Study. Pathobiology.

[33] Subbiah V., Kreitman R.J., Wainberg Z.A., Cho J.Y., Schellens J.H.M., Soria J.C., Wen P.Y., Zielinski C., Cabanillas M.E., Urbanowitz G. (2018). Dabrafenib and Trametinib Treatment in Patients with Locally Advanced or Metastatic BRAF V600-Mutant Anaplastic Thyroid Cancer. J. Clin. Oncol..

[34] Bible K.C., Kebebew E., Brierley J., Brito J.P., Cabanillas M.E., Clark T.J., Di Cristofano A., Foote R., Giordano T., Kasperbauer J. (2021). 2021 American Thyroid Association Guidelines for Management of Patients with Anaplastic Thyroid Cancer. Thyroid.

[35] Baloch Z.W., Asa S.L., Barletta J.A., Ghossein R.A., Juhlin C.C., Jung C.K., LiVolsi V.A., Papotti M.G., Sobrinho-Simoes M., Tallini G. (2022). Overview of the 2022 WHO Classification of Thyroid Neoplasms. Endocr. Pathol..

[36] Saglietti C., Piana S., La Rosa S., Bongiovanni M. (2017). Hyalinizing trabecular tumour of the thyroid: Fine-needle aspiration cytological diagnosis and correlation with histology. J. Clin. Pathol..

[37] Nikiforova M.N., Nikiforov Y.E., Ohori N.P. (2019). GLIS rearrangements in thyroid nodules: A key to preoperative diagnosis of hyalinizing trabecular tumor. Cancer Cytopathol..

[38] Agrawal N., Akbani R., Aksoy B.A., Ally A., Arachchi H., Asa S.L., Auman J.T., Balasundaram M., Balu S., Baylin S.B. (2014). Integrated genomic characterization of papillary thyroid carcinoma. Cell.

[39] Fagin J.A., Wells S.A. (2016). Biologic and Clinical Perspectives on Thyroid Cancer. N. Engl. J. Med..

[40] Landa I., Ibrahimpasic T., Boucai L., Sinha R., Knauf J.A., Shah R.H., Dogan S., Ricarte-Filho J.C., Krishnamoorthy G.P., Xu B. (2016). Genomic and transcriptomic hallmarks of poorly differentiated and anaplastic thyroid cancers. J. Clin. Investig..

[41] Pozdeyev N., Gay L.M., Sokol E.S., Hartmaier R., Deaver K.E., Davis S., French J.D., Borre P.V., LaBarbera D.V., Tan A.C. (2018). Genetic Analysis of 779 Advanced Differentiated and Anaplastic Thyroid Cancers. Clin. Cancer Res..

[42] Ibrahimpasic T., Ghossein R., Shah J.P., Ganly I. (2019). Poorly Differentiated Carcinoma of the Thyroid Gland: Current Status and Future Prospects. Thyroid.

[43] Erdian D.N., Ham M.F., Khoirunnisa D., Harahap A.S. (2025). High Ki-67 labeling index correlates with aggressive clinicopathological features in papillary thyroid carcinoma: A retrospective study. Thyroid Res..

[44] Ahn S., Kim T.H., Kim S.W., Ki C.S., Jang H.W., Kim J.S., Kim J.H., Choe J.H., Shin J.H., Hahn S.Y. (2017). Comprehensive screening for PD-L1 expression in thyroid cancer. Endocr.-Relat. Cancer.

[45] Montemayor-Garcia C., Hardin H., Guo Z., Larrain C., Buehler D., Asioli S., Chen H., Lloyd R.V. (2013). The role of epithelial mesenchymal transition markers in thyroid carcinoma progression. Endocr. Pathol..

[46] Hanna M.G., Reuter V.E., Hameed M.R., Tan L.K., Chiang S., Sigel C., Hollmann T., Giri D., Samboy J., Moradel C. (2019). Whole slide imaging equivalency and efficiency study: Experience at a large academic center. Mod. Pathol..

[47] Kim J., Kim M.H., Lim D.J., Lee H., Lee J.J., Kwon H.S., Kim M.K., Song K.H., Kim T.J., Jung S.L. (2025). Deep Learning Technology for Classification of Thyroid Nodules Using Multi-View Ultrasound Images: Potential Benefits and Challenges in Clinical Application. Endocrinol. Metab..

[48] Lee Y., Alam M.R., Park H., Yim K., Seo K.J., Hwang G., Kim D., Chung Y., Gong G., Cho N.H. (2024). Improved Diagnostic Accuracy of Thyroid Fine-Needle Aspiration Cytology with Artificial Intelligence Technology. Thyroid.

[49] Tao Y., Yu Y., Wu T., Xu X., Dai Q., Kong H., Zhang L., Yu W., Leng X., Qiu W. (2022). Deep learning for the diagnosis of suspicious thyroid nodules based on multimodal ultrasound images. Front. Oncol..

[50] Chen X., Zhang L., Chen B., Lu J. (2025). Building radiomics models based on ACR TI-RADS combining clinical features for discriminating benign and malignant thyroid nodules. Front. Endocrinol..

[51] Wei R., Wang H., Wang L., Hu W., Sun X., Dai Z., Zhu J., Li H., Ge Y., Song B. (2021). Radiomics based on multiparametric MRI for extrathyroidal extension feature prediction in papillary thyroid cancer. BMC Med. Imaging.

[52] Lang M., Longerich T., Anamaterou C. (2023). Targeted therapy with vemurafenib in BRAF(V600E)-mutated anaplastic thyroid cancer. Thyroid Res..

[53] Wirth L.J., Sherman E., Robinson B., Solomon B., Kang H., Lorch J., Worden F., Brose M., Patel J., Leboulleux S. (2020). Efficacy of Selpercatinib in RET-Altered Thyroid Cancers. N. Engl. J. Med..

[54] Drilon A., Laetsch T.W., Kummar S., DuBois S.G., Lassen U.N., Demetri G.D., Nathenson M., Doebele R.C., Farago A.F., Pappo A.S. (2018). Efficacy of Larotrectinib in TRK Fusion-Positive Cancers in Adults and Children. N. Engl. J. Med..

[55] Filetti S., Durante C., Hartl D., Leboulleux S., Locati L.D., Newbold K., Papotti M.G., Berruti A. (2019). Thyroid cancer: ESMO Clinical Practice Guidelines for diagnosis, treatment and follow-up†. Ann. Oncol..

